# Sudden Death in Young Brazilian Athletes: Isn't It Time We Created a Genuinely National Register?

**DOI:** 10.5935/abc.20180207

**Published:** 2018-12

**Authors:** Lucas Helal, Filipe Ferrari, Ricardo Stein

**Affiliations:** 1 Programa de Pós-graduação em Cardiologia e Ciências Cardiovasculares da Faculdade de Medicina da Universidade Federal do Rio Grande do Sul, Porto Alegre, RS - Brasil; 2 Laboratório de Fisiopatologia do Exercício, Hospital de Clínicas de Porto Alegre, Porto Alegre, RS - Brazil; 3 Grupo de Pesquisa em Cardiologia do Exercício, Hospital de Clínicas de Porto Alegre, Porto Alegre, RS - Brazil; 4 Departamento de Medicina Interna da Faculdade de Medicina da Universidade Federal do Rio Grande do Sul, Porto Alegre, RS - Brazil

**Keywords:** Cardiovascular Diseases, Athletes, Adolescent, Youth Sports, Death, Sudden, Cardiac, Genotype

## Abstract

Young competitive athletes (≤ 35 years old) with or without a previous diagnosis of cardiovascular disease may suddenly die in competitive activities, potentially leading to an impact in society through the media. Although the relative risk for sudden death (SD) in athletes is twice as high as for their counterparts, the absolute incidence is low. While there is consensus among medical societies worldwide that early detection of causal factors is highly desirable, there is debate among different screening schemes to that end. In Brazil, the recommendations of the Brazilian Society of Cardiology mirror the guidelines of the European Society of Cardiology (ESC), which indicate a clinical examination combined with a 12-lead resting electrocardiogram, regardless of the presence of risk factors. The possibility of genetic screening is also plausible, since most clinical entities that cause SD in young competitive athletes are related to genotype. Finally, considering the diversity of practiced sports, and the population miscegenation, we emphasize the need to a national registry of cases.

## Introduction

Sudden death (SD) in young athletes (under 35 years old) is a peculiar event. Despite being rare, cases have been reported by far-reaching media, which may cause a major impact on both health agencies and the society. The counterintuitive feeling that young, presumably asymptomatic individuals with above-average physical fitness may die suddenly during sports practice seems particularly striking to many people, especially when it affects elite athletes.

People involved in intense competitive activities have a relative SD risk that is nearly twice as high as that of their non-athlete counterparts, though incidence in absolute numbers is very low - 0.5 to 2 events per 100,000 athletes per year.^1^ Usually, clinical entities of cardiac or vascular nature, whether previously diagnosed or not, are the most prevalent causes of SD, and participation in sports events one of is the triggering factor for its occurrence. The most frequent are of genetic origin and hereditary, whether they structural (e.g., myocardiopathies) or not (e.g., channelopathies). On the other hand, the aortic (e.g., Marfan Syndrome) and coronary artery diseases are less prevalent but have also been described in this age group.^2^ Based on evidence unrelated to exercise, external causal factors can also be considered. For example, the use of central nervous system stimulant drugs and anabolic steroids seems to increase the risk of SD in adults,^3,4^ so it is plausible to hypothesize that athletes exposed to these risk factors may add to that statistic.

In relation to its prevention, some success rate can be achieved if the disease is detected in time. There is treatment available for some illnesses and, on certain occasions, there may be a medical decision to suspend the athlete's participation in competitive sport (disqualification), thereby protecting them. Therefore, whenever possible, early detection of triggering diseases should be made. However, medical societies in various countries recommend different screening schemes.^5,6^ Since the etiology of causative factors can differ regarding geography, ethnicity, sporting modality, genetic inheritance, and age, this point-of-view article aims to discuss the need for a national register of cases so the best prevention and early detection strategy may be laid out in Brazil - an idea already mentioned in this journal seven years ago.^7^

### Screening Schemes Suggested and the Absence of a Brazilian Register

In 2011, Peidro, Froelicher and Stein published a point of view discussing particularities of Pre-Participation Physical Examination (PPE) for SD prevention in young athletes in Argentina and Brazil.^7^ At the time, given the experiences that the American and Italian communities had about the effectiveness of the different decision algorithms, the need for national registers (in Argentina and Brazil) for SD cases was suggested. Since then, little has been done, and the national recommendation is not based on local data or robust evidence, such as: a) the prevalence of SD and its causes; b) ideally, subsequent evaluation of the effectiveness of potential screening strategies by means of randomized clinical trials; c) health technology assessment the algorithm to be proposed.

The Brazilian Society of Cardiology (SBC) and the Brazilian Society of Exercise and Sports Medicine (SBMEE) jointly recommend the same screening scheme as the European Society of Cardiology (ESC)^6,8^ - i.e., anamnesis, physical examination and 12-lead resting electrocardiogram (ECG), regardless of the presence or absence of risk factors. The scheme proposed by the ESC is heavily influenced by observational evidence collected in Italy. Initially, it was found that young athletes from the Veneto region were at high risk of SD during competitive sports practice, compared to other regions of the world.^9^ In 2006, Corrado et al.^10^ highlighted a high prevalence of Arrhythmogenic right ventricular cardiomyopathy (ARVC) and hypothesized that disqualifying athletes with a diagnosis or suspicion of that disease (or similar diseases) would be effective to reduce SD mortality at sports events.^10^ These findings increased the discussion on the implementation of a specific algorithm that would make ECG mandatory in addition to anamnesis and physical examination as screening procedures for anyone engaging in a competitive and structured physical exercise program. Two years later and criticism aside, a classic study published by the same group of researchers tested the scheme and demonstrated it reduced SD cases by 89% in competitive activities involving young athletes in that country.^11^ In fact, the PPE has been mandatory for more than two decades for every regulated amateur or professional athlete, with an Italian federal law that is adopted by all sports regulation entities.^12^

On the other hand, the joint procedure recommended by the American College of Cardiology (ACC) and the American College of Sports Medicine (ACSM) do not include a mandatory ECG, as argue that the annual incidence of SD in the United States is much lower than in Italy.^5^ In addition, they reiterate that ECG's sub-optimal specificity for detecting anomalies in athletes may result in an excessive number of false-positive results.

In this regard, the overall consequences of a false-positive result^13^ may lead to unnecessary over-investigation (e.g., echocardiography, cardiac resonance, among other examinations), with undesired financial and personal costs, or even disqualifications. However, contrary to the arguments of the American entities above, a cost-effectiveness analysis conducted by a Stanford group^14^ pointed out that including ECG in the clinical examination would prevent 2.09 additional deaths per 1,000 athletes, with an estimated individual cost of U$ 89 per examination and a cost-effectiveness estimate of approximately U$ 43,000 per qualitiy-adjusted life years (QALY).

For Brazil, these data are particularly important, since the Ministry of Health is currently discussing, together with the National Congress and academic entities involved in the assessment of health technologies, the threshold of willingness to pay for added technology. Because until the present the cost per QALY of a new technology is unknown, we believe it is critical that decisions be made in Brazil based on knowledge of local statistics, which again reinforces the need for a national register of SD in young athletes to be properly conducted.

### Why a National Register is Necessary

Because the etiology of SD in sports in young athletes is diverse, with regional and genetic influences, identifying the local prevalence is of utmost importance to make decisions based on evidence. For example, ethnicity has an effect on the incidence of SD in young athletes. In the US, an increased incidence of SD was found in black basketball and football players compared with other ethnic groups, most often due to hypertrophic cardiomyopathy (HCM).^15^ Autopsy data from that country showed that that twice as many black athletes died from HCM as white athletes (20% vs. 10%).^16^ Such information suggests a possible divergence in HCM presentation in different ethnic groups and that it may be more malignant in black individuals.

In fact, in at least 50% of the cases, HCM presents as an autosomal dominant monogenic disease. Its overall prevalence is traditionally estimated at 1:500 individuals,^17^ but more recent data indicate that it may be even more frequent than previously established, which is corroborated by advances in genetic research.^18^ In Brazil, the frequency of HCM is not solidly known. Here, it is our opinion that this disease is an example of the importance of ECG in the context of competitive athletes, as it can be suspected through this examination in a high percentage of cases. Moreover, changes in the ST segment, in the T wave, as well as the presence of pathological Q waves^19^ can be warning signs based on which further exams (with a higher positive predictive value) can be requested in a context of greater pretest probability.

Once there is a diagnosis (clinical and/or molecular), septal ablation, myectomy, and/or an implantable cardioverter defibrillator (ICD) may be indicated. Likewise, but only in well-selected cases, disqualification may be the final outcome for the athlete's career.^20,21^ In respect to the ethnicity of the Brazilian population, which is extremely mixed and with a high prevalence of blacks,^22^ knowing the causes of SD in Brazilian athletes of this race seems to us very important to classify the risk for these individuals.

As for the causes of SD due to ion channel disturbance, genetic screening,^23^ already included in the SBC's 2013 guidelines,^8^ has been suggested as a possible strategy to help with prevention (note of the authors: when properly indicated), given the high contribution of the genetic factor for the event's occurrence.^24^ given the high contribution of the genetic factor for the event's occurrence.^24^ In fact, some malignant mutations are already known in genes that cause Long QT Syndrome (LQTS), Short QT Syndrome, Brugada Syndrome and catecholaminergic polymorphic ventricular tachycardia (CPVT).^25,26^ Here, we would like to point out that although molecular mapping is accessible and technical improvement through new generation sequencing is available in our country, requesting it is still not part of our medical culture, even in more robust clinical scenarios (e.g., breast neoplasm screening and association with BRCA genes).^27^ In fact, in order for medical entities to formally encourage its request in PPE, it is necessary to know prevalence so that the technology can be submitted to the health technology assessment (HTA) process, which is similar to the processes already performed for other clinical entities.^28^

The sport modality also has some bearing on the construction of a decision algorithm for PPE and SD prevention in young athletes. For example, in the United States, SD prevalence is higher in basketball and football.^29^ In Europe, SD in young athletes is more frequent in field soccer players.^30^ As a matter of curiosity, in combat sports, which involve high-energy trauma, or in sports involving interaction with high-speed artifacts (e.g., baseball), concerns with commotio cordis (a malignant arrhythmia triggered by direct trauma in the anterior thorax) must be present.^31^

### Future Directions

**Figure 1:**
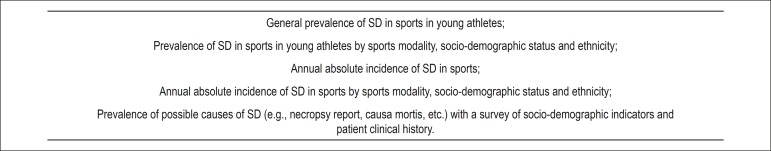
Suggestion of statistics to be surveyed by means of a national register of SD cases in competitive sports in young athletes. SD: sudden death.

As exposed above, we identified a few points still unknown which require priority examination in order to define tracking, prevention and national regulation strategies, all of which are essential for the HTA process; and which can be accessed through a genuinely Brazilian register (Figure 1).

We emphasize the central role of public research support agencies in Brazil for building a register. A call for projects specifically to that end seems to us appropriate, preferably for projects of a multicentric nature and with public sharing of data. So far, considering the lack interest on the part of independent researchers, the State has not yet expressed a position, nor has it facilitated the implementation of a viable strategy for a national register, which we consider a critical step.

## References

[1] Maron BJ (2003). Sudden death in young athletes. N Engl J Med.

[2] Maron BJ, Epstein SE, Roberts WC (1986). Causes of sudden death in competitive athletes. J Am Coll Cardiol.

[3] Morentin B, Ballesteros J, Callado LF, Meana JJ (2014). Recent cocaine use is a significant risk factor for sudden
cardiovascular death in 15-49-year-old subjects a forensic case-control
study. Addiction.

[4] Frati P, Busardo FP, Cipolloni L, Dominicis ED, Fineschi V (2015). Anabolic Androgenic Steroid (AAS) related deaths autoptic,
histopathological and toxicological findings. Curr Neuropharmacol.

[5] Maron BJ, Levine BD, Washington RL, Baggish AL, Kovacs RJ, Maron MS (2015). Eligibility and disqualification recommendations for competitive
athletes with cardiovascular abnormalities Task Force 2: Preparticipation
Screening for Cardiovascular Disease in Competitive Athletes: A Scientific
Statement From the American Heart Association and American College of
Cardiology. Circulation.

[6] Corrado D, Pelliccia A, Heidbuchel H, Sharma S, Link M, Basso C (2010). Recommendations for interpretation of 12-lead electrocardiogram
in the athlete. Eur Heart J.

[7] Peidro R, Froelicher V, Stein R (2011). Pre-participation screening of the young athlete is this the time
for an agreement?. Arq Bras Cardiol.

[8] Monteiro VCL, Silva JAdO, Oliveira RB, Frangipani BJ, Dearo PR, Previdelli ÁN (2018). Evaluation of food intake in patients with
mucopolysaccharidosis. Nutrire Rev Soc Bras Aliment Nutr.

[9] Pelliccia A, Maron BJ (1995). Preparticipation cardiovascular evaluation of the competitive
athlete Perspectives from the 30-year Italian experience. Am J Cardiol.

[10] Corrado D, Basso C, Pavei A, Michieli P, Schiavon M, Thiene G (2006). Trends in sudden cardiovascular death in young competitive
athletes after implementation of a preparticipation screening
program. JAMA.

[11] Corrado D, Basso C, Schiavon M, Pelliccia A, Thiene G (2008). Pre-participation screening of young competitive athletes for
prevention of sudden cardiac death. J Am Coll Cardiol.

[12] Lakhdar N, Denguezli M, Zaouali M, Zbidi A, Tabka Z, Bouassida A (2014). Six months training alone or combined with diet alters HOMA-AD,
HOMA-IR and plasma and adipose tissue adiponectin in obese
women. Neuro Endocrinol Lett.

[13] Grimes DA, Schulz KF (2002). Uses and abuses of screening tests. Lancet.

[14] Wheeler MT, Heidenreich PA, Froelicher VF, Hlatky MA, Ashley EA (2010). Cost-effectiveness of preparticipation screening for prevention
of sudden cardiac death in young athletes. Ann Intern Med.

[15] Maron BJ, Carney KP, Lever HM, Lewis JF, Barac I, Casey SA (2003). Relationship of race to sudden cardiac death in competitive
athletes with hypertrophic cardiomyopathy. J Am Coll Cardiol.

[16] Maron BJ, Doerer JJ, Haas TS, Tierney DM, Mueller FO (2009). Sudden deaths in young competitive athletes analysis of 1866
deaths in the United States, 1980-2006. Circulation.

[17] Maron BJ, Maron MS (2013). Hypertrophic cardiomyopathy. Lancet.

[18] Semsarian C, Ingles J, Maron MS, Maron BJ (2015). New perspectives on the prevalence of hypertrophic
cardiomyopathy. J Am Coll Cardiol.

[19] Kelly BS, Mattu A, Brady WJ (2007). Hypertrophic cardiomyopathy electrocardiographic manifestations
and other important considerations for the emergency
physician. Am J Emerg Med.

[20] Elliott PM, Anastasakis A, Borger MA, Borggrefe M, Cecchi F, Charron P (2014). 2014 ESC Guidelines on diagnosis and management of hypertrophic
cardiomyopathy the Task Force for the Diagnosis and Management of
Hypertrophic Cardiomyopathy of the European Society of Cardiology
(ESC). Eur Heart J.

[21] Gersh BJ, Maron BJ, Bonow RO, Dearani JA, Fifer MA, Link MS (2011). 2011 ACCF/AHA guideline for the diagnosis and treatment of
hypertrophic cardiomyopathy executive summary: a report of the American
College of Cardiology Foundation/American Heart Association Task Force on
Practice Guidelines. Circulation.

[22] Olesen J, Ringholm S, Nielsen MM, Brandt CT, Pedersen JT, Halling JF (2013). Role of PGC-1alpha in exercise training- and resveratrol-induced
prevention of age-associated inflammation. Exp Gerontol.

[23] Ackerman MJ, Priori SG, Willems S, Berul C, Brugada R, Calkins H (2011). HRS/EHRA expert consensus statement on the state of genetic
testing for the channelopathies and cardiomyopathies this document was
developed as a partnership between the Heart Rhythm Society (HRS) and the
European Heart Rhythm Association (EHRA). Europace.

[24] Garfinkel AC, Seidman JG, Seidman CE (2018). Genetic Pathogenesis of Hypertrophic and Dilated
Cardiomyopathy. Heart Fail Clin.

[25] Fernandez-Falgueras A, Sarquella-Brugada G, Brugada J, Brugada R, Campuzano O (2017). Cardiac channelopathies and sudden death: recent clinical and
genetic advances. Biology (Basel).

[26] Crotti L, Kotta MC (2017). The role of genetics in primary ventricular fibrillation,
inherited channelopathies and cardiomyopathies. Int J Cardiol.

[27] Ewald IP, Cossio SL, Palmero EI, Pinheiro M, Nascimento IL, Machado TM (2016). BRCA1 and BRCA2 rearrangements in Brazilian individuals with
Hereditary Breast and Ovarian Cancer Syndrome. Genet Mol Biol.

[28] Ingles J, McGaughran J, Scuffham PA, Atherton J, Semsarian C (2012). A cost-effectiveness model of genetic testing for the evaluation
of families with hypertrophic cardiomyopathy. Heart.

[29] Maron BJ, Shirani J, Poliac LC, Mathenge R, Roberts WC, Mueller FO (1996). Sudden death in young competitive athletes Clinical, demographic,
and pathological profiles. JAMA.

[30] Higgins JP, Andino A (2013). Soccer and sudden cardiac death in young competitive athletes a
review. J Sports Med (Hindawi Publ Corp).

[31] Maron BJ, Gohman TE, Kyle SB, Estes 3rd NA, Link MS (2002). Clinical profile and spectrum of commotio cordis. JAMA.

